# Seroprevalence and Risk Factors for *Toxoplasma gondii* Infection in Horses

**DOI:** 10.3390/vetsci10030237

**Published:** 2023-03-22

**Authors:** Mohamed Marzok, Omar A. AL-Jabr, Mohamed Salem, Khalid Alkashif, Mohamed Sayed-Ahmed, Majed H. Wakid, Mahmoud Kandeel, Abdelfattah Selim

**Affiliations:** 1 Department of Clinical Sciences, College of Veterinary Medicine, King Faisal University, Al-Hofuf 31982, Saudi Arabia; 2 Department of Surgery, Faculty of Veterinary Medicine, Kafr El Sheikh University, Kafr El Sheikh 33511, Egypt; 3 King Faisal University Veterinary Hospital, Al-Asha 31982, Saudi Arabia; 4 Department of Microbiology, College of Veterinary Medicine, King Faisal University, Al-Asha 31982, Saudi Arabia; 5 Department of Medicine and Infectious Diseases, Faculty of Veterinary Medicine, Cairo University, Cairo 12613, Egypt; 6 Department of Pharmacology and Toxicology, College of Pharmacy, Jazan University, Jazan 82722, Saudi Arabia; 7 Department of Clinical Pharmacy, College of Pharmacy, Jazan University, Jazan 82722, Saudi Arabia; 8 Department of Internal Medicine and Infectious Diseases, Faculty of Veterinary Medicine, Mansoura University, Mansoura 35516, Egypt; 9 Department of Medical Laboratory Technology, Faculty of Applied Medical Sciences, King Abdulaziz University, Jeddah 21589, Saudi Arabia; 10 Special Infectious Agents Unit, King Fahd Medical Research Center, King Abdulaziz University, Jeddah 21589, Saudi Arabia; 11 Department of Pharmacology, Faculty of Veterinary Medicine, Kafr El Sheikh University, Kafr El Sheikh 33511, Egypt; 12 Department of Biomedical Sciences, College of Veterinary Medicine, King Faisal University, Al-Ahsa 31982, Saudi Arabia; 13 Department of Animal Medicine (Infectious Diseases), Faculty of Veterinary Medicine, Benha University, Toukh 13736, Egypt

**Keywords:** horse, indirect ELISA, *T. gondii*, seroprevalence, risk factor, Egypt

## Abstract

**Simple Summary:**

*Toxoplasma gondii* is classified as intracellular protozoa and is one of the major zoonotic parasites. Mares, horses of mixed breeds, and older than five years are substantially more likely to contract *T. gondii* infection. In addition, horses raised in contact with cats or domestic ruminants are more likely to test positive for *T. gondii* infection. This study confirms that horses in Northern Egypt are exposed to *T. gondii* and raises the possibility that people and other animals could contract the disease.

**Abstract:**

Background: *Toxoplasma gondii* is classified as intracellular protozoa and is one of the major zoonotic parasites. Most warm-blooded intermediate hosts, including humans, are commonly infected by this parasite. The epidemiology of *T. gondii* infection in Egyptian horses is currently poorly understood. Methods: 420 blood samples were randomly collected from horses raised in four governorates in Northern Egypt (110 each from Giza and Kafr El Sheikh, and 100 each from Qalyubia and Gharbia) to investigate the existence of antibodies against *T. gondii* using a commercial ELISA kit, and to ascertain the risk factors for the infection. Results: the antibodies for *T. gondii* were found in 16.2% (68/420) of the examined horses, with no significant differences among the four studied governorates. The highest prevalence rate was observed in Giza. The results revealed that sex, breed, age, and contact with domestic ruminants or cats were recognized as potential risk factors. The high prevalence rate was found in mixed breed horses (OR = 2.63, 95% CI: 0.95–7.26), mares (OR = 2.35, 95% CI: 1.31–4.19), and horses aged over 10 years (OR = 2.78, 95% CI: 1.30–3.44). Moreover, the likelihood of seropositivity for *T. gondii* infection was higher in horses raised in environments with cats (OR = 1.97, 95% CI: 1.13–3.44, *p* = 0.017) or domestic ruminants (OR = 2.16, 1.21–3.86, *p* = 0.010). This report confirms that horses in Northern Egypt are exposed to *T. gondii* and thus raises the possibility that people and other animals could contract the disease. Conclusions: routine examination and management of *T. gondii* infection in horses in these governorates is advised.

## 1. Introduction

*Toxoplasma gondii* is classified as a globally prevalent intracellular protozoan, which causes toxoplasmosis [1,2,3]. Domestic cats and other felids are the definitive hosts for *T. gondii* [4]. Only these hosts can release oocysts into the environment, which can contaminate pastures, food, and water. Around 100 million non-sporulated oocysts can be passed by a single cat, and these oocysts become infective between one and five days later [5]. Nevertheless, the parasite has a variety of intermediate hosts, including mammals, such as humans, and birds that harbour the cyst stage in their tissues [6,7]. 

*T. gondii* infection in herbivores and equines mostly happens through water or food contaminated with sporulated oocysts, however, transplacental transmission of tachyzoites from mother to foetus is also possible [8,9].

Toxoplasmosis is a chronic disease causing cystic formation in the tissues of the hosts [10], however, the life cycle of *T. gondii* depends on oocysts, which become infectious to a wide range of warm-blooded intermediate hosts if consumed after one to a few days of maturation (sporulation) in the environment. Oocysts are not the only infective stages of *T. gondii*; there are also tachyzoites and bradyzoites, the latter of which is seen in tissue cysts. Tachyzoites infiltrate host cells after infection and grow there. When the parasite forms parasitophorous vacuoles, this replication is strictly intracytoplasmic. Parallel to this, the parasite creates internal tissue cysts that contain bradyzoites that are no longer multiplying or are only replicating slowly after multiple rounds of multiplication [11]. The infected intermediate hosts’ brain tissue, skeletal and cardiac muscles, and even their retinas, are the preferred locations for tissue cyst formation. The parasite has a complicated life cycle, and there are numerous ways for infection [12]. 

The majority of infections in people are asymptomatic, but congenital *Toxoplasma* infections can have serious side effects, including stillbirth, abortion, mortality, and hydrocephalus in newborns, as well as retinochoroidal lesions that cause retinitis, chronic ocular disease, lymphadenopathy, and encephalitis in people with compromised immune systems [8].

Since it was originally isolated in 1908, *T. gondii* has spread globally. In northern Iraq, a serosurvey of sheep and goats revealed seroprevalences of *Toxoplasma* infection of 42.1% and 36.1%, respectively [13]. In addition, *T. gondii* infection in canine was detected in Brazil and revealed a seropositivity of 9.54% [14]. The seroprevalence of *T. gondii* in animals ranged from 6% to 33%, according to statistical analysis of the positive rates of *T. gondii* infection in five different animal species in the Nordic-Baltic region [15]. *T. gondii* infection may affect one-third of the global population according to the Centers for Disease Control and Prevention (CDC), *T. gondii* infects approximately 22.5% of Americans over 12 years old in the United States, although recent years have seen a slight reduction in prevalence [16].

In Egypt, anti-*T. gondii* antibodies were found in 10.8% of cattle based on ELISA test results [17] and in 43.7% or 41.7% of sheep examined by a modified agglutination test or ELISA [18], respectively. Furthermore, donkeys and horses have high corresponding seroprevalences of 65.6% and 48.1%, respectively [19,20].

*T. gondii* is a significant contributor to small ruminant reproductive problems, resulting in abortion, stillbirth, mummification, infertility, and the delivery of poor lambs [21,22]. Although *T. gondii* infection in horses is typically asymptomatic [23], fever, abortion, degeneration in retina, and stillbirth were recorded in infected pregnant mares [24,25].

Moreover, one of the main ways that humans become infected is through the ingestion of undercooked, contaminated meat, although eating horse meat is not common for people in Egypt [1], and eating horse meat from the Americas has been epidemiologically associated to serious sickness in Europeans [26,27]. *Toxoplasma* infection can also develop through other means, such as eating undercooked, contaminated meat (particularly lamb, pork, and venison) or shellfish (such as clams or mussels) [28]. Because humans can become infected by consuming contaminated meat, *T. gondii* infection not only results in financial and reproductive losses but also has an impact on public health [12,29].

The incidence of *T. gondii* in human across Egypt is not well-documented. The majority of serological reports are founded on convenience samples, including those taken from pregnant women and patients with illnesses. Moreover, *T. gondii* DNA was discovered in 10% (15/150) of Alexandria blood donors [30] and in 6% of the blood donors from the Qalubiya governorate [31].

A common reference test for the serosurvey of toxoplasmosis in various animal species is the latex agglutination test (LAT) [32]. However, the ELISA test displays greater potency, sensitivity, and specificity when compared to other reference serodiagnostic tests, such as the LAT, the modified agglutination test, the direct agglutination test, and the indirect fluorescent antibody test, which are used to identify anti-*T. gondii* antibodies in serum samples from various animals [33,34,35].

Although monitoring of *T. gondii* infection is crucial to prevent its proliferation, Egypt has limited information on its prevalence in horses [19,36]. Understanding the frequency of *T. gondii* in horses is vital to prevent infection through consumption of contaminated meat, as Egypt’s carnivorous zoo animals are routinely given raw horse meat [36].

Consequently, the purpose of this study was to evaluate the risk factors for *T. gondii* infection in horses in four governorates of northern Egypt and to ascertain the seroprevalence of *T. gondii* therein.

## 2. Material and Methods

### 2.1. Ethical Statement

Benha University’s Animal Ethics Committee approved this work (Approval no. BUFVTM02-10-22, Benha). Serum samples were obtained and handled in conformity with the Animal Ethics Procedures and Guidelines of the Committee. This study was conducted according to ARRIVE guidelines. In this study, we worked on live horses and we did not anesthetize and/or sacrifice those animals.

### 2.2. Study Area

**Figure 1:**
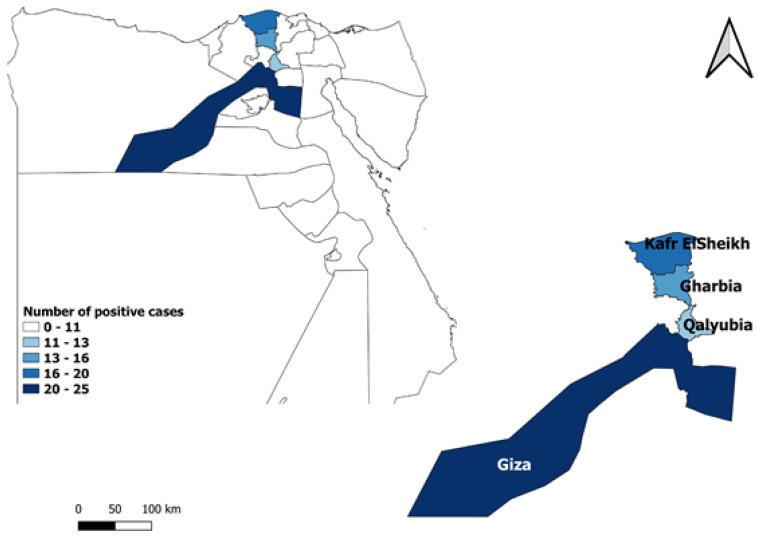
Map showing four the Egyptian governorates where horses were sampled (map generated by QGIS software 3.18.3).

The study was conducted in northern Egypt, specifically in the governorates of Giza, Kafr El Sheikh, Qalyubia, and Gharbia, which are geographically situated at 29°59′13.2″ N 31°12′42.48″ E, 31°06′42″ N 30°56′45″ E, 30°24′36″ N 31°12′36″ E, 30°52′1.2″ N 31°1′40.8″ E, respectively (Figure 1). The large equine population in these regions led to their selection for this research.

Egypt has a predominantly hot, arid environment (Köppen climatic classification BWh). The country’s climate is often rather dry, except the northern Mediterranean coast. The climate in Giza is arid. Summers are quite warm and winters are mild in this type of environment. Here, rain rarely falls.

The other governorates are situated in the Nile Delta of Egypt. The climate of these areas, much like the rest of Egypt, is a hot desert climate (Köppen: BWh), but its northernmost area, like the rest of Egypt’s northern coast, which is the country’s wettest region, has more moderate temperatures, with summer highs typically not exceeding 31 °C. On average, the delta region receives only 100–200 mm (4–8 in) of rain each year, with the majority of this precipitation occurring in the winter.

### 2.3. Sample Collection and Preparation

A cross-sectional study was conducted between January and December of 2020. By using simple random sampling, blood samples from horses were obtained.

On the basis of the 48.1% prevalence rate of *T. gondii* in horses in Egypt as reported by Ghazy, et al. [20], the predicted prevalence was set as 48.1% (*P*), with a 5% (*d*) absolute precision and a 95% confidence level (z = 1.96). The sample size of 420 exceeds the minimum sample size (383) according to the following formula: N = *P* (1 − *P*)z^2^/d^2^ [37].

Blood samples were taken from horses raised in the four selected areas. Each animal had a jugular vein venipuncture, and sterile 10-mL tubes free of anticoagulant were used to collect 5 mL of blood from each horse. The blood samples were labelled, preserved in an icebox, and then transported to the Veterinary Diagnostic Laboratory at the Faculty of Veterinary Medicine at Benha University. Prior to further serological investigation, the sera were separated by centrifugation for 10 min at 3000 xg, and then stored in 1.5 mL tubes at −20 °C.

At the time of sample collection, the data of each animal was collected through a questionnaire filled in by the owner or veterinarian. According to collected data, animals were categorised according to region (Giza, Kafr El Sheikh, Qalyubia, or Gharbia), breed (Arabian, thoroughbred, or mixed), sex (male or female), and age groups (<5, 5–10, or >10 years old). The questionnaire also included questions about farm biosecurity, such as if cats or other domestic ruminants were present. The details of examined horses are presented in Table 1. All of the horses under investigation were fed wheat bran, some green grass, and agricultural waste items, such as barley and maize. In addition, water was provided three times per day.

### 2.4. Serological Examination

Horse serum samples were examined using the commercial kit ID Screen^®^ Toxoplasmosis Indirect Multi-species Indirect ELISA (ID Vet, Montpellier, France) for *T. gondii* immunoglobulin G (IgG) antibodies. The kit uses a multi-species peroxidase as a conjugate and the P30 *T. gondii* protein as a substrate to detect specific IgG antibodies following *T. gondii* infection.

The testing was performed in accordance with the manufacturer’s recommendations, and the optical densities (ODs) of the ELISA findings were read at 450 nm. Each test serum’s sample (*S*) to positive (*P*) ratio (*S*/*P*%) was determined using the following formula:

$$\frac{S}{P}\%=\frac{OD_{sample}-OD_{negative\;control}}{OD_{positive\;control}-OD_{negative\;control}}\times 100$$

According to the manufacturer guidelines, samples with *S*/*P*% readings above 50% were regarded as positive, below 40% were considered negative and those between 40 to 50% considered doubtful.

### 2.5. Statistical Analysis

The SPSS software version24.0 (IBM, New York, NY, USA) was used for data analysis. The differences in the seroprevalences of variable categories were determined by Chi-square test, with *p*-values of <0.05 considered as significant. The relationship between *T. gondii* infection and the relevant risk variables was examined using univariate analysis. Multivariate logistic regression models were then fitted for all variables in the univariate analysis with a *p*-value less than 0.25. To determine the degree of relation between the presence of *T. gondii* and variables, the odds ratios (ORs) and 95% confidence intervals (95% CIs) were calculated. The model fit was assessed using the Hosmer and Lemeshow goodness-of-fit test.

## 3. Results 

*T. gondii* had a 16.2% seroprevalence in the examined horses, with 68 samples from 420 horses testing positive for *T. gondii* via an ELISA test. *T. gondii* antibodies were detected in samples from all four governorates, with 20.9% in Giza, 17.3% in Kafr El Sheikh, 12% in Qalyubia and 14% in Gharbia. The difference was statistically non-significant (*p* = 0.31), Table 2.

Interestingly, statistically significant differences (*p* < 0.05) occurred in the seroprevalence of *T. gondii* between horse breeds and sexes. *T. gondii* seroprevalence was higher in females (20.9%) than in males (10.5%), and higher in mixed breed (21.3%) than in Arabian (8.3%) and thoroughbred horses (14.5%), as shown in Table 3.

Furthermore, *T. gondii* seroprevalence was 22.7% in horses older than 10 years, a value which was significantly higher than the rates of other age groups (16.7% for horses aged 5 to 10 years and 10% for those under 5 years; *p* = 0.02). The results highlight the fact that higher *T. gondii* seroprevalence (*p* < 0.05) was found in horses raised in contact with cats (21.1%) or domestic ruminants (23.3%) (Table 3).

Multivariate analysis results in Table 4 established that the variables of breed, sex, age, presence of cats, and presence of domestic ruminants, were all independently associated with *T. gondii* infection in horses in Egypt. The risk for mares was 2.35 times (95% CI: 1.31–4.19) greater than for male horses. Mixed breed horses were had 2.63 times (95% CI: 0.95–7.26) greater risk than their Arabian counterparts. Further, horses aged 5–10 years have a 2.23 times (95% CI: 1.06–4.70) greater risk, and those >10 years have a 2.78 times (95% CI: 1.30–5.95) greater risk, than those younger than 5 years old. Both the presence of domestic ruminants (OR = 2.16, 95% CI: 1.21–3.86, *p* = 0.010) and cats (OR = 1.97, 95% CI: 1.13–3.44, *p* = 0.017) in close proximity to horses increased the risk of *T. gondii* seroprevalence in horses, Table 4.

## 4. Discussion 

*T. gondii* is one of the most significant zoonotic pathogens, as *T. gondii* infection in horses has become one of the most significant possible causes of human toxoplasmosis [1]. Unfortunately, little information is available about the epidemiology of *T. gondii* infection in horses in Egypt, and the majority of existing research is outdated [19,20]. The present study aimed to determine the seroprevalence of *T. gondii* in horses in northern Egypt using the ELISA technique, as well as to ascertain the risk factors for *T. gondii* infection.

In this work, *T. gondii* seroprevalence was 16.2% in horses, a value which is lower than previous reported rate (25%) in Egypt by Haridy, et al. [38], but is close to value of (17.92%) observed in northern Chinese horses [39]. However, the seroprevalence rates in the present study were lower than those reported by Almeida et al. [40] (23.64% in Brazil); Saqib et al. [41] (23.50% in Pakistan); Bártová et al. [42] (24.17% in Nigeria); Razmi et al. [43] (20.30% in Iran); and Alanazi and Alyousif [44] (31.58% in Saudi Arabia). By contrast, the seroprevalence rates of this work were higher than those reported by Aharonson-Raz et al. [45] (3.25% in Israel); García-Bocanegra et al. [23] (10.79% in Spain); Lopes et al. [46] (13.29% in Portugal); Boughattas et al. [47] (17.72% in Tunisia); and Karatepe et al. [48] (7.20% in Turkey). Moreover, relatively lower seroprevalence levels were found in Sweden (0.5–1%) [49] and Greece (1.8%) [50] and relatively higher values were found in Italy (30.7%) [51], in numerous studies in Turkey (20.6–28%) [52,53] and in recently conducted studies in the Czech Republic (23%) [54].

The following factors may be responsible for the variations in seroprevalence: time of sampling, sample size, differences in the sensitivity of the detection methods, the cut off titer utilised in the interpretation of findings, animal susceptibility, the number and age of examined horses, location, feeding practices, sanitation, and farming management [23,39,40,55,56,57,58,59]. Additionally, climate-linked influences that include regional distribution, density of population, and the presence or absence of cats or of animals that serve as reservoir hosts or transport hosts, are crucial to the emergence, survival, dispersion, and transmission of *T. gondii* [3,60,61,62,63,64,65].

This work did not detect significant differences in *T. gondii* seroprevalence according to sampling location, an outcome similar to that of a study from Japan [10]. Nevertheless, *T. gondii* seroprevalence varied in the examined areas, being notably higher in the Giza governorate in comparison to other locations. This may be due to the Giza governorate’s very hospitable climate for the growth of oocysts, especially its humidity and temperature, as well as the governorate’s substantial animal populations that are bred in big groups.

There is currently no proof that *T. gondii* infects horses spontaneously or through experimental exposure to induce clinical illness [66]. Nonetheless, live *T. gondii* has been identified from naturally exposed horses [36], and people in France who ate raw imported horse meat had severe clinical toxoplasmosis. According to findings obtained by Shaapan and Ghazy [36], the prevalence of *T. gondii* in Egypt is very high. Moreover, they isolated viable *T. gondii* by mouse bioassay in 79 of 150 pools of tissue samples from 150 horses slaughtered in the Giza Zoo abattoir. As a result, eating horseflesh could potentially infect humans as well as captive felines in zoos. *T. gondii*-infected horsemeat should not be offered to cats or consumed by humans, as a study shown that it may survive in edible tissues of living horses for up to 476 days [66]. Hence, equids might be involved in the spread of *T. gondii* to humans and cats in Egypt.

Furthermore, *T. gondii* seroprevalence varied significantly amongst equine breeds. El-Ghaysh [19] suggested that being kept as free-ranging animals explained the higher seroprevalence of *T. gondii* among mixed breeds and thoroughbreds relative to the Arabian counterpart. That is, the non-Arabian breeds may come into contact with oocysts in the contaminated environment more frequently. These findings were consistent with those of García-Bocanegra, et al. [23] who found that seroprevalence was higher in crossbred horses than in stabled thoroughbreds because the former frequently roam more freely and have greater access to parasites. Finally, the type of activity and location have been proven to exert a substantial impact on the presence of *T. gondii* infection in horses in Egypt [36,67,68,69,70,71]. 

The available data about the seroprevalences of *T. gondii* in other equids than horses are very little. The few published studies on donkeys establish the high seroprevalence of *T. gondii* in Egypt, at a range between 45% and 66% [19,72]. Mules, which are more closely related to donkeys than horses, demonstrated intermediate *T. gondii* levels between that of horses and donkeys, an outcome which is consistent with findings from China (8.6% seroprevalence in mules against 4.5% in horses) [72].

The present findings support the previous observation of Haridy, et al. [38] which indicates that females have more contact with contaminated environments than males, but contradict the outcomes from previous studies in Tunisia and Turkey [47,48]. Also, it implies that female horses are more susceptible to parasite infection than male and gelding horses, which is consistent with the findings of an earlier study conducted in Egypt [38]. Similar findings were also found in Spain [23], northwest Algeria [73], and Xinjiang, northwestern China [74], however unlike our study, their difference was not demonstrated to be statistically significant.

In line with earlier findings, younger horses have a lower seroprevalence, and statistically significant differences occur between age groups [50]. This result implies that equids contract an infection during their first few years of life and that the exposure remains continuous as they age, thereby explaining the higher prevalence of *T. gondii* infection in older horses [47,74,75,76]. By contrast, other research revealed a negative relationship between age and *T. gondii* infection in horses as well as a high prevalence rate in younger horses, the latter of which may be mostly due to underdeveloped immune systems [39] or as a result of trans-placental transmission and horizontal infection of *T. gondii* resulting from the consumption of oocyst-contaminated food or water. In addition, recent research from other nations, in contrast to our findings, revealed no appreciable variation in the seroprevalence of *T. gondii* in horses from Mexico, southern Spain, Korea, and southern Italy within age classes [23,77,78,79,80].

Equines and other intermediate hosts for *T. gondii*, such as ruminants, can only get infected after eating or drinking products contaminated with sporulated *T. gondii* oocysts from cats, or through congenital transmission [1,2]. High seroprevalence has been seen in felid species in the past, including domestic cats (50%) and Iberian lynx (*Lynx pardinus*) (62.8%) in Andalusia [81,82]. *T. gondii* oocysts are believed to have been released into the environment by seropositive felids. 

In addition, it has been noted that the presence of cats is one of the major contributors to *T. gondii* seroprevalence in other domestic animals, including pigs [82] and small ruminants [83]. It should be highlighted that the present findings may have been impacted by the challenge of accurately estimating the amount of cats in shelters or even pastures where horses are housed.

The present work confirmed that raising horses in contact with cats was a risk factor for *T. gondii* infection, which is consistent with findings of previous studies [4,13,47]. The variations in the environment’s level of contamination by cats, which are the definitive hosts of *T. gondii*, and other mechanical carriers of oocysts, such as rodents, may be the cause of the disparities in seroprevalence.

The presence of domestic ruminants was associated with the seroprevalence of *T. gondii* in equids as determined in a study in Spain [23]. In Egypt, *T. gondii* is regarded as one of the major causes of small ruminant abortion and neonatal death. In Egypt, research on *T. gondii* seroprevalence in domestic ruminants revealed significant antibody prevalence levels in cattle (10.8%), sheep (98.4%), goats (41.7%), and camels (46.9%) [3,35]. 

## 5. Conclusions

This study confirmed that *T. gondii* circulates in the horse populations in four governorates in Egypt, thereby posing a concern to the health of both animals and people. In addition, breed, sex, age, the presence of cats, and the presence of domestic ruminants, are identified as risk factors for the infection. These results are intended to provide Egyptian authorities with helpful information for managing and preventing toxoplasmosis in horses and/or other hosts. Finally, further seroepidemiological studies are necessary to investigate the prevalence of *T. gondii* in wider areas in Egypt, and to identify the epidemiological situation of the parasite across the country.

## Figures and Tables

**Table 1:** Descriptive analysis of the factors used to determine the seroprevalence of *T. gondii* infection in Egyptian horses.

Variable	Category	No. of Horses	Distribution
**Locality**	Giza	110	26.2%
	Kafr El Sheikh	110	26.2%
	Qalyubia	100	23.8%
	Gharbia	100	23.8%
**Breed**	Arabian	60	14.3%
	Thoroughbred	200	47.6%
	Mixed	160	38.1%
**Sex**	Male	190	45.2%
	Female	230	54.7%
**Age**	<5 year	130	31%
	5–10	180	42.8%
	>10	110	26.2%
**Presence of cats**	Yes	180	42.9%
	No	240	57.1%
**Presence of domestic ruminants**	Yes	120	28.6%
	No	300	71.4%

**Table 2:** Seroprevalence of *T. gondii* in horses from different governorates under the study.

Factor	No. of Examined Horses	No. of Positive	% of Positive	95% CI	*p*-Value
**Locality**					
Giza	110	23	20.9	14.36–29.43	
Kafr El Sheikh	110	19	17.3	11.34–25.41	
Qalyubia	100	12	12.0	7–19.81	0.31
Gharbia	100	14	14.0	8.53–22.14	

**Table 3:** Univariate analysis of the association of focal variables with seropositivity to *T. gondii* in horses (N = 420) in Egypt.

Factor	No. of Examined Horses	No. of Positive	% of Positive	95% CI	Statistics
**Breed**					
Arabian	60	5	8.3	3.61–18.06	χ2 = 6.167 df = 2 *p* = 0.04 *
Thoroughbred	200	29	14.5	10.29–20.05	
Mixed	160	34	21.3	15.62–28.22	
**Sex**					
Male	190	20	10.5	6.92–15.7	χ2 = 8.203 df = 1 *p* = 0.004 *
Female	230	48	20.9	16.12–26.58	
**Age**					
<5 years	130	13	10.0	5.94–16.36	χ2 = 7.165 df = 2 *p* = 0.02 *
5–10 years	180	30	16.7	11.93–22.8	
>10 years	110	25	22.7	15.9–31.4	
**Presence of cats**					
Yes	180	38	21.1	15.78–27.64	χ2 = 5.621 df = 1 *p* = 0.02 *
No	240	30	12.5	8.9–17.28	
**Presence of domestic ruminants**					
Yes	120	28	23.3	16.66–31.65	χ2 = 6.317 df = 1 *p* = 0.01 *
No	300	40	13.3	9.94–17.64	
Total	420	68	16.2	12.98–20.02	

* The result considered significant at *p* < 0.05.

**Table 4:** Final multivariate mode of the risk factors for *Toxoplasma gondii* infection in horses in Egypt.

Variable	B	S.E.	OR	95% CI for OR		*p*-Value
				Lower	Upper	
**Breed**						
Thoroughbred	0.574	0.519	1.78	0.64	4.90	0.268
Mixed	0.965	0.519	2.63	0.95	7.26	0.063
**Sex**						
Female	0.852	0.296	2.35	1.31	4.19	0.004
**Age**						
5–10 years	0.803	0.380	2.23	1.06	4.70	0.034
>10 years	1.023	0.388	2.78	1.30	5.95	0.008
**Presence of cats**						
Yes	0.679	0.283	1.97	1.13	3.44	0.017
**Presence of domestic ruminants**						
Yes	0.769	0.297	2.16	1.21	3.86	0.010

B—logistic regression coefficient; SE—standard error; OR—odds ratio; CI—confidence interval.

## Data Availability

This article contains all of the data that was created or analyzed throughout the investigation.

## References

[1] Dubey J.P. (2016). Toxoplasmosis of Animals and Humans.

[2] Innes E. (2010). A brief history and overview of *Toxoplasma gondii*. Zoonoses Public Health.

[3] Selim A., Marawan M.A., Abdelhady A., Wakid M.H. (2023). Seroprevalence and Potential Risk Factors of *Toxoplasma gondii* in Dromedary Camels. Agriculture.

[4] Dabritz H., Conrad P.A. (2010). Cats and *Toxoplasma*: Implications for public health. Zoonoses Public Health.

[5] Schlüter D., Däubener W., Schares G., Groß U., Pleyer U., Lüder C. (2014). Animals are key to human toxoplasmosis. Int. J. Med. Microbiol..

[6] Tong W.H., Pavey C., O’Handley R., Vyas A. (2021). Behavioral biology of *Toxoplasma gondii* infection. Parasites Vectors.

[7] Yang S., Parmley S.F. (1997). *Toxoplasma gondii* expresses two distinct lactate dehydrogenase homologous genes during its life cycle in intermediate hosts. Gene.

[8] Hill D., Dubey J. (2002). *Toxoplasma gondii*: Transmission, diagnosis and prevention. Clin. Microbiol. Infect..

[9] Tassi P. (2007). *Toxoplasma gondii* infection in horses. A review. Parassitologia.

[10] Masatani T., Takashima Y., Takasu M., Matsuu A., Amaya T. (2016). Prevalence of anti-*Toxoplasma gondii* antibody in domestic horses in Japan. Parasitol. Int..

[11] Stelzer S., Basso W., Silván J.B., Ortega-Mora L.M., Maksimov P., Gethmann J., Conraths F., Schares G. (2019). *Toxoplasma gondii* infection and toxoplasmosis in farm animals: Risk factors and economic impact. Food Waterborne Parasitol..

[12] Dubey J.P., Murata F., Cerqueira-Cézar C., Kwok O. (2020). Public health and economic importance of *Toxoplasma gondii* infections in goats: The last decade. Res. Vet. Sci..

[13] Al Hamada A., Habib I., Barnes A., Robertson I. (2019). Risk factors associated with seropositivity to *Toxoplasma* among sheep and goats in Northern Iraq. Vet. Parasitol. Reg. Stud. Rep..

[14] Souza I.B.d., Fernandes P.R., Silva T.R.M., Santos C.V.B., Silva N.M.M.d., Ubirajara Filho C.R.C., Carvalho G.A.d., Alves L.C., Mota R.A., Ramos R.A.N. (2019). Seroprevalence of *Neospora caninum* and *Toxoplasma gondii* in dogs from an urban area of North-eastern Brazil: A spatial approach. Rev. Da Soc. Bras. De Med. Trop..

[15] Olsen A., Berg R., Tagel M., Must K., Deksne G., Enemark H.L., Alban L., Johansen M.V., Nielsen H.V., Sandberg M. (2019). Seroprevalence of *Toxoplasma gondii* in domestic pigs, sheep, cattle, wild boars, and moose in the Nordic-Baltic region: A systematic review and meta-analysis. Parasite Epidemiol. Control..

[16] Guo M., Dubey J.P., Hill D., Buchanan R.L., Gamble H.R., Jones J.L., Pradhan A.K. (2015). Prevalence and risk factors for *Toxoplasma gondii* infection in meat animals and meat products destined for human consumption. J. Food Prot..

[17] Ibrahim H.M., Huang P., Salem T.A., Talaat R.M., Nasr M.I., Xuan X., Nishikawa Y. (2009). Prevalence of *Neospora caninum* and *Toxoplasma gondii* antibodies in northern Egypt. Am. J. Trop. Med. Hyg..

[18] Shaapan R., El-Nawawi F., Tawfik M. (2008). Sensitivity and specificity of various serological tests for the detection of *Toxoplasma gondii* infection in naturally infected sheep. Vet. Parasitol..

[19] El-Ghaysh A. (1998). Seroprevalence of *Toxoplasma gondii* in Egyptian donkeys using ELISA. Vet. Parasitol..

[20] Ghazy A., Shaapan R., Abdel-Rahman E.H. (2007). Comparative serological diagnosis of toxoplasmosis in horses using locally isolated *Toxoplasma gondii*. Vet. Parasitol..

[21] Dubey J., Murata F., Cerqueira-Cézar C., Kwok O., Su C. (2020). Economic and public health importance of *Toxoplasma gondii* infections in sheep: 2009–2020. Vet. Parasitol..

[22] Kakakhel M.A., Wu F., Anwar Z., Saif I., ul Akbar N., Gul N., Ali I., Feng H., Wang W. (2021). The presence of *Toxoplasma gondii* in soil, their transmission, and their influence on the small ruminants and human population: A review. Microbial. Pathogenesis.

[23] García-Bocanegra I., Cabezón O., Arenas-Montes A., Carbonero A., Dubey J., Perea A., Almería S. (2012). Seroprevalence of *Toxoplasma gondii* in equids from Southern Spain. Parasitol. Int..

[24] Gazyağci S., Macun H., Babür C. (2011). Investigation of seroprevalance of toxoplasmosis in mares and stallions in Ankara province, Turkey. Iran. J. Vet. Res..

[25] Cazarotto C.J., Balzan A., Grosskopf R.K., Boito J.P., Portella L.P., Vogel F.F., Fávero J.F., Cucco D.d.C., Biazus A.H., Machado G. (2016). Horses seropositive for *Toxoplasma gondii*, *Sarcocystis* spp. and *Neospora* spp.: Possible risk factors for infection in Brazil. Microb. Pathog..

[26] Aroussi A., Vignoles P., Dalmay F., Wimel L., Dardé M.-L., Mercier A., Ajzenberg D. (2015). Detection of *Toxoplasma gondii* DNA in horse meat from supermarkets in France and performance evaluation of two serological tests. Parasite.

[27] Alvarado-Esquivel C., Alvarado-Esquivel D., Dubey J.P. (2015). Prevalence of *Toxoplasma gondii* antibodies in domestic donkeys (*Equus asinus*) in Durango, Mexico slaughtered for human consumption. BMC Vet. Res..

[28] Dixon B., Fayer R., Santín M., Hill D., Dubey J. (2011). Protozoan parasites: *Cryptosporidium, Giardia, Cyclospora,* and *Toxoplasma*. Rapid Detect. Charact. Enumer. Foodborne Pathog..

[29] Shwab E.K., Saraf P., Zhu X.-Q., Zhou D.-H., McFerrin B.M., Ajzenberg D., Schares G., Hammond-Aryee K., van Helden P., Higgins S.A. (2018). Human impact on the diversity and virulence of the ubiquitous zoonotic parasite *Toxoplasma gondii*. Proc. Natl. Acad. Sci. USA.

[30] El-Geddawi O.A., El-Sayad M.H., Sadek N.A., Hussien N.A., Ahmed M.A. (2016). Detection of *T. gondii* infection in blood donors in Alexandria, Egypt, using serological and molecular strategies. Parasitol. United J..

[31] El-Sayed N.M., Abdel-Wahab M.M., Kishik S.M., Alhusseini N.F. (2016). Do we need to screen Egyptian voluntary blood donors for toxoplasmosis?. Asian Pac. J. Trop. Dis..

[32] Matsuo K., Kamai R., Uetsu H., Goto H., Takashima Y., Nagamune K. (2014). Seroprevalence of *Toxoplasma gondii* infection in cattle, horses, pigs and chickens in Japan. Parasitol. Int..

[33] Terkawi M.A., Kameyama K., Rasul N.H., Xuan X., Nishikawa Y. (2013). Development of an immunochromatographic assay based on dense granule protein 7 for serological detection of *Toxoplasma gondii* infection. Clin. Vaccine Immunol..

[34] Gu Y., Wang Z., Cai Y., Li X., Wei F., Shang L., Li J., Liu Q. (2015). A comparative study of *Toxoplasma gondii* seroprevalence in mink using a modified agglutination test, a Western blot, and enzyme-linked immunosorbent assays. J. Vet. Diagn. Investig..

[35] Fereig R.M., Mahmoud H.Y., Mohamed S.G., AbouLaila M.R., Abdel-Wahab A., Osman S.A., Zidan S.A., El-Khodary S.A., Mohamed A.E.A., Nishikawa Y. (2016). Seroprevalence and epidemiology of *Toxoplasma gondii* in farm animals in different regions of Egypt. Vet. Parasitol. Reg. Stud. Rep..

[36] Shaapan R., Ghazy A. (2007). Isolation of *Toxoplasma gondii* from horse meat in Egypt. Pak. J. Biol. Sci. PJBS.

[37] Daniel W.W., Cross C.L. (2018). Biostatistics: A Foundation for Analysis in the Health Sciences.

[38] Haridy F.M., Shoukry N.M., Hassan A.A., Morsy T.A. (2009). ELISA-seroprevalence of *Toxoplasma gondii* in draught horses in Greater Cairo, Egypt. J. Egypt. Soc. Parasitol..

[39] Zhang X.-X., Ren W.-X., Hou G., Liu Q., Yu T.-Q., Zhao Q., Ni H.-B. (2018). Seroprevalence and risk factors of *Toxoplasma gondii* infection in horses in Jilin Province and Inner Mongolia Autonomous Region, Northern China. Acta Tropica.

[40] Almeida J.C., Vidotto O., Ferreira E.P., Ribeiro L.P., Mongruel A.C., Vieira T.S., Freire R.L., Mota R.A., Vieira R.F. (2017). Serosurvey of anti-*Toxoplasma gondii* antibodies in sport horses from Paraiba state, Northeastern Brazil. Acta Parasitol..

[41] Saqib M., Hussain M., Sajid M., Mansoor M., Asi M., Fadya A., Zohaib A., Sial A., Muhammad G., Ullah I. (2015). Sero-epidemiology of equine toxoplasmosis using a latex agglutination test in the three metropolises of Punjab, Pakistan. Trop. Biomed..

[42] Bártová E., Sedlák K., Kobédová K., Budíková M., Atuman Y.J., Kamani J. (2017). Seroprevalence and risk factors of *Neospora* spp. and *Toxoplasma gondii* infections among horses and donkeys in Nigeria, West Africa. Acta Parasitol..

[43] Razmi G.R., Abedi V., Yaghfoori S. (2016). Serological study of *Toxoplasma gondii* infection in Turkoman horses in the North Khorasan Province, Iran. J. Parasit. Dis..

[44] Alanazi A.D., Alyousif M.S. (2011). Prevalence of antibodies to *Toxoplasma gondii* in horses in Riyadh Province, Saudi Arabia. J. Parasitol..

[45] Aharonson-Raz K., Baneth G., Lopes A.P., Brancal H., Schallig H., Cardoso L., Steinman A. (2015). Low seroprevalence of *Leishmania infantum* and *Toxoplasma gondii* in the horse population in Israel. Vector-Borne Zoonotic Dis..

[46] Lopes A.P., Sousa S., Dubey J., Ribeiro A.J., Silvestre R., Cotovio M., Schallig H.D., Cardoso L., Cordeiro-da-Silva A. (2013). Prevalence of antibodies to *Leishmania infantum* and *Toxoplasma gondii* in horses from the north of Portugal. Parasites Vectors.

[47] Boughattas S., Bergaoui R., Essid R., Aoun K., Bouratbine A. (2011). Seroprevalence of *Toxoplasma gondii* infection among horses in Tunisia. Parasites Vectors.

[48] Karatepe B., Babür C., Karatepe M., Kılıç S. (2010). Seroprevalence of toxoplasmosis in horses in Niğde Province of Turkey. Trop. Anim. Health Prod..

[49] Uggla A., Mattson S., Juntti N. (1990). Prevalence of antibodies to *Toxoplasma gondii* in cats, dogs and horses in Sweden. Acta Vet. Scand..

[50] Kouam M.K., Diakou A., Kanzoura V., Papadopoulos E., Gajadhar A.A., Theodoropoulos G. (2010). A seroepidemiological study of exposure to *Toxoplasma, Leishmania, Echinococcus* and *Trichinella* in equids in Greece and analysis of risk factors. Vet. Parasitol..

[51] Rinaldi L., Scala A. (2008). Toxoplasmosis in livestock in Italy: An epidemiological update. Parassitologia.

[52] AKCA A., Babur C., ARSLAN M., Gicik Y., Kara M., KILIC S. (2004). Prevalence of antibodies to *Toxoplasma gondii* in horses in the province of Kars, Turkey. Vet. Med..

[53] Güçlü Z., Karaer Z., Babür C., Kiliç S. (2007). Investigation of *Toxoplasma gondii* antibodies in sport horses bred in Ankara province. Positivity.

[54] Bártová E., Sedlák K., Syrová M., Literák I. (2010). *Neospora* spp. and *Toxoplasma gondii* antibodies in horses in the Czech Republic. Parasitol. Res..

[55] Meng Q.-F., Li D., Yao G.-Z., Zou Y., Cong W., Shan X.-F. (2018). Seroprevalence of *Toxoplasma gondii* infection and variables associated with seropositivity in donkeys in eastern China. Parasite.

[56] Selim A., Abdelhady A. (2020). The first detection of anti-West Nile virus antibody in domestic ruminants in Egypt. Trop. Anim. Health Prod..

[57] Selim A., Ali A.-F., Ramadan E. (2019). Prevalence and molecular epidemiology of Johne’s disease in Egyptian cattle. Acta tropica.

[58] Selim A., Attia K., Ramadan E., Hafez Y.M., Salman A. (2019). Seroprevalence and molecular characterization of *Brucella* species in naturally infected cattle and sheep. Prev. Vet. Med..

[59] Selim A., Khater H., Almohammed H.I. (2021). A recent update about seroprevalence of ovine neosporosis in Northern Egypt and its associated risk factors. Sci. Rep..

[60] Prestrud K.W., Åsbakk K., Fuglei E., Mørk T., Stien A., Ropstad E., Tryland M., Gabrielsen G.W., Lydersen C., Kovacs K.M. (2007). Serosurvey for *Toxoplasma gondii* in arctic foxes and possible sources of infection in the high Arctic of Svalbard. Vet. Parasitol..

[61] Dhimal M., Ahrens B., Kuch U. (2014). Species composition, seasonal occurrence, habitat preference and altitudinal distribution of malaria and other disease vectors in eastern Nepal. Parasites Vectors.

[62] Selim A., Ali A.-F., Moustafa S.M., Ramadan E. (2018). Molecular and serological data supporting the role of Q fever in abortions of sheep and goats in northern Egypt. Microb. Pathog..

[63] Selim A., Manaa E., Khater H. (2021). Seroprevalence and risk factors for lumpy skin disease in cattle in Northern Egypt. Trop. Anim. Health Prod..

[64] Selim A., Manaa E.A., Alanazi A.D., Alyousif M.S. (2021). Seroprevalence, risk factors and molecular identification of bovine leukemia virus in Egyptian cattle. Animals.

[65] Selim A., Manaa E.A., Waheed R.M., Alanazi A.D. (2021). Seroprevalence, associated risk factors analysis and first molecular characterization of *chlamydia abortus* among Egyptian sheep. Comp. Immunol. Microbiol. Infect. Dis..

[66] Dubey J. (1985). Persistence of encysted *Toxoplasma gondii* in tissues of equids fed oocysts. Am. J. Vet. Res..

[67] Selim A., Megahed A., Kandeel S., Alouffi A., Almutairi M.M. (2021). West Nile virus seroprevalence and associated risk factors among horses in Egypt. Sci. Rep..

[68] Selim A., Megahed A.A., Kandeel S., Abdelhady A. (2020). Risk factor analysis of bovine leukemia virus infection in dairy cattle in Egypt. Comp. Immunol. Microbiol. Infect. Dis..

[69] Selim A., Yang E., Rousset E., Thiéry R., Sidi-Boumedine K. (2018). Characterization of *Coxiella burnetii* strains from ruminants in a *Galleria mellonella* host-based model. New Microbes New Infect..

[70] Selim A.M., Elhaig M.M., Gaede W. (2014). Development of multiplex real-time PCR assay for the detection of *Brucella* spp., *Leptospira* spp. and *Campylobacter foetus*. Vet. Ital..

[71] Selim A.M., Elhaig M.M., Moawed S.A., El-Nahas E. (2018). Modeling the potential risk factors of bovine viral diarrhea prevalence in Egypt using univariable and multivariable logistic regression analyses. Vet. World.

[72] Ling C., Wan P. (1984). A report of investigations of antibodies to *Toxoplasma gondii* in the horse and mule in Sichuan Province. Zhongguo Shouyi Ke-Ji.

[73] Cherif M., Ait Oudhia K., Khelef D. (2015). Detection of anti-Toxoplasma gondiiantibodies among horses (*Equus caballus*) and donkeys (*Equus asinus*) in Tiaret province, northwestern Algeria. Revue Méd. Vét..

[74] Wang J.-L., Zhou D.-H., Chen J., Liu G.-X., Pu W.-B., Liu T.-Y., Qin S.-Y., Yin M.-Y., Zhu X.-Q. (2015). The prevalence of antibodies to *Toxoplasma gondii* in horses in Changji Hui Autonomous Prefecture, Xinjiang, northwestern China. Rev. Bras. De Parasitol. Veterinária.

[75] Li X., Ni H.-B., Ren W.-X., Jiang J., Gong Q.-L., Zhang X.-X. (2020). Seroprevalence of *Toxoplasma gondii* in horses: A global systematic review and meta-analysis. Acta Trop..

[76] Tavalla M., Sabaghan M., Abdizadeh R., Khademvatan S., Rafiei A., Piranshahi A.R. (2015). Seroprevalence of *Toxoplasma gondii* and *Neospora* spp. infections in Arab horses, southwest of Iran. Jundishapur J. Microbiol..

[77] Ouslimani S.F., Tennah S., Azzag N., Derdour S.Y., China B., Ghalmi F. (2019). Seroepidemiological study of the exposure to *Toxoplasma gondii* among horses in Algeria and analysis of risk factors. Vet. World.

[78] Alvarado-Esquivel C., García-Machado C., Alvarado-Esquivel D., Vitela-Corrales J., Villena I., Dubey J. (2012). Seroprevalence of *Toxoplasma gondii* infection in domestic sheep in Durango State, Mexico. J. Parasitol..

[79] Lee S.-H., Lee S.-E., Seo M.-G., Goo Y.-K., Cho K.-H., Cho G.-J., Kwon O.-D., Kwak D., Lee W.-J. (2014). Evidence of *Toxoplasma gondii* exposure among horses in Korea. J. Vet. Med. Sci..

[80] Bártová E., Machaèová T., Sedlák K., Budíková M., Mariani U., Veneziano V. (2015). Seroprevalence of antibodies of *Neospora* spp. and *Toxoplasma gondii* in horses from southern Italy. Folia Parasitol..

[81] Millán J., Candela M.G., Palomares F., Cubero M.J., Rodríguez A., Barral M., de la Fuente J., Almería S., León-Vizcaíno L. (2009). Disease threats to the endangered Iberian lynx (*Lynx pardinus*). Vet. J..

[82] García-Bocanegra I., Dubey J., Martínez F., Vargas A., Cabezón O., Zorrilla I., Arenas A., Almería S. (2010). Factors affecting seroprevalence of *Toxoplasma gondii* in the endangered Iberian lynx (*Lynx pardinus*). Vet. Parasitol..

[83] Mainar R., De La Cruz C., Asensio A., Domínguez L., Vázquez-Boland J. (1996). Prevalence of agglutinating antibodies to *Toxoplasma gondii* in small ruminants of the Madrid region, Spain, and identification of factors influencing seropositivity by multivariate analysis. Vet. Res. Commun..

